# Saudi adolescents’ perceptions of dietary contributions to obesity: A qualitative study

**DOI:** 10.1371/journal.pone.0334407

**Published:** 2025-11-14

**Authors:** Nasser S. Alqahtani

**Affiliations:** Community Health Department, Northern Border University, Arar, Saudi Arabia; Yonsei University Medical Center: Yonsei University Health System, KOREA, REPUBLIC OF

## Abstract

**Background:**

Adolescents’ perceptions of the dietary effects of obesity play an imporant part in shaping their eating behaviors, lifestyle choices, and overall health outcomes. Their beliefs and attitudes surrounding diet and obesity can significantly influence food intake, nutritional habits, and levels of physical activity. This study aimed to examine adolescents’ perceptions of how diet contributes to obesity.

**Materials and methods:**

This qualitative, prospective observational study included 62 adolescents of 12‒18 years old who were categorized as overweight or obese. Selection of participants was through purposive convenience sampling. Data were gathered through interviews and analyzed using Microsoft Office Excel tools.

**Results:**

Five primary themes emerged from the analysis: (1) determinants of obesity, (2) strategies for obesity prevention, (3) educational and health system factors, (4) stigma associated with being overweight, and (5) perceived health risks due to obesity. The most cited factor contributing to obesity was the widespread availability of unhealthy food. Participants also noted weak school policies, experiences of being stigmatized (e.g., teasing and diminished self-esteem), and concerns about the health risks associated with obesity. Prevention strategies emphasized the importance of motivation, awareness, and institutional support.

**Conclusion:**

Adolescents identified the accessibility of fast food and a lack of knowledge as major contributors to obesity. Weak school and health policies were also noted as contributing factors. Being stigmatized, particularly in the form of mocking, emerged as the most prominent social challenge. Participants believed obesity increases disease risks and highlighted individual motivation as important in prevention efforts. The findings highlight the need to promote healthy habits and strengthen awareness among adolescents.

## Introduction

The rising incidence of obesity has become a critical public health concern globally. Individuals across all age groups and socioeconomic strata are affected [1]. One hallmark characteristic of this challenge is the widespread prevalence of excess weight and obesity, with the World Health Organization (WHO) reporting that, worldwide, 36.9% of men and 38.0% of women are overweight or obese [1]. This trend persists across both developed and developing nations and has shown no signs of slowing. Particularly alarming are the disparities in youth obesity rates: in developed countries, 23.8% of boys and 22.6% of girls are considered overweight or obese, whereas in dveloping countres 12.9% of boys and 13.4% of girls are considered overweight or obese [2]. These early-onset weight challenges set the stage for future health complications, including diabetes, cardiovascular diseases, and mental health disorders [3].

Adolescence represents a critical window for shaping lifelong health behaviors. During this period, hormonal changes, body image sensitivity, and peer influence converge to form deeply embedded habits. Research shows that many adolescents experience distorted perceptions of their own body weight, which can contribute to disordered eating, sedentary lifestyles, and chronic stress [4,5]. For instance, Brechan and Kvalem highlight how dissatisfaction with body image is often linked to both low self-esteem and depressive symptoms, especially in girls [4]. Similarly, Becker et al. found that adolescents with weight concerns were significantly more likely to engage in binge eating, restrictive dieting, and use of unhealthy weight control methods [6]. These behaviors are often driven less by actual body mass and more by perceived weight, suggesting the psychological lens through which adolescents interpret their bodies has powerful behavioral consequences [6].

Tong et al., in a longitudinal cohort study, revealed that adolescents who misperceived themselves as overweight had nearly double the risk of becoming obese in young adulthood, independent of their baseline BMI [7]. However, while such findings underscore the importance of self-perception, the literature remains inconsistent when it comes to how weight perception shapes specific behaviors like diet quality or physical activity [8]. This gap is especially notable in non-Western contexts, where sociocultural factors such as gender norms, religious expectations (e.g., fasting during Ramadan), and regional/traditional family food patterns interact with environmental influences to shape adolescent attitudes toward weight and health [9].

For example, Cauchi et al. reported that Kiribati high school students identified lack of access to nutritious food, low participation in physical activity, and minimal school-based health instruction as key contributors to obesity [10]. Their findings demonstrated strong associations between obesity and both meal skipping and reduced sports participation (OR = 2.4 and OR = 3.12, respectively), reinforcing the need for institutional interventions. Tong et al., in follow-up research, further emphasized that addressing adolescent obesity requires more than individual behavior change; it demands policy-level reforms that incorporate health education into curricula and increase opportunities for physical activity [11]. While these findings offer valuable insights, the Saudi Arabian context remains underexplored, particularly regarding how rapid modernization [12], shifts in food availability [13], and gender-segregated schooling influence adolescents’ dietary perceptions and behaviors [14].

Given the multifactorial nature of adolescent obesity, solutions must be multifaceted. Research after 2000 calls for a broader understanding of how cultural beliefs, environmental exposures, and institutional support interact to shape youth perceptions and behaviors [15]. The concept of *nutritional transition*, defined as the shift from traditional diets high in whole grains, legumes, and fiber toward energy-dense, processed foods high in sugar and saturated fats, often accompanied by reduced physical activity, provides a helpful lens for examining these changes [12,16]. In Saudi Arabia, this transition has been accelerated by urbanization, globalization, and changing lifestyle patterns, creating new challenges for adolescent health [16].

Recognizing these complex dynamics, the present study aims to explore how Saudi adolescents perceive the dietary effects of obesity within their local context. Through in-depth, face-to-face interviews, this research seeks to uncover the beliefs, attitudes, and lived experiences that shape dietary behaviors and body image perceptions in a society undergoing rapid nutritional and cultural transitions.

## Methodology

### Study design and setting

The study employed a prospective, observational qualitative design to explore adolescents’ perceptions of dietary factors contributing to overweight and obesity. Participants were recruited between 28/01/2025 and 22/03/2025. The research was conducted across multiple rural and suburban regions in Saudi Arabia over nine weeks. Data were gathered using in-depth, face-to-face interviews conducted in Arabic, permitting participants to share their personal experiences and insights in a culturally relevant context.

### Sampling and selection bias mitigation

A purposive convenience sampling strategy was used to recruit overweight or obese adolescents aged 12–18 years. Participants were selected to reflect diversity in gender, geographic location, and school type. Adolescents who participated in the pilot study or declined consent were excluded. To minimize selection bias, the researcher collaborated with school administrators and community health centers to ensure a varied and representative sample. Recruitment continued until thematic saturation was achieved, which was determined when no new codes or subthemes emerged after eight interviews, consistent with the guidance of Sandelowski [17].

### Quantification of dietary and lifestyle context

Although this was a qualitative study, key lifestyle indicators (e.g., screen time, meal frequency, and food environment) were explored through structured prompts during interviews. Participants were asked about their typical food choices, physical activity routines, and frequency of meals. This contextual information supported the identification and interpretation of qualitative themes and mirrored efforts made in other mixed-method adolescent obesity research [18].

### Rationale for interview methods

In-depth, face-to-face interviews were chosen to elicit a nuanced understanding of participants’ beliefs, motivations, and perceptions and enabled individualized exploration of sensitive topics such as body image, stigma, and lifestyle behaviors. It also allowed for clarification of ambiguous responses and the adaptation of follow-up questions to suit participant comfort and comprehension.

### Preparation of interview guide

The interview guide comprised six open-ended questions aligned with the study’s objectives. Questions were developed by reviewing existing literature and studies conducted in similar contexts [11,17,19]. Experts in adolescent health reviewed the guide, and the guide underwent pilot testing for clarity, cultural appropriateness, and flow. For transparency, a summary of the six main questions is provided in S1 File. Interviews were conducted in Arabic and translated into English for analysis. The researcher and an accredited translator validated translations.

### Study sample

School principals and community health centers were approached to grant access and permission for the distribution of invitation letters, detailed information sheets, and assent and consent forms to potential participants within the eligible age range (12‒18 years). Participants came from various regions across Saudi Arabia and attended both public and private schools. Schools lacking access to adequate facilities for private interviewing were excluded.

Selection criteria included adolescents aged 12–18 with an overweight or obese status based on the Body Mass Index (BMI). Being overweight was defined as a BMI at or above the 85th percentile but below the 95th percentile for age and sex, while obesity was defined as a BMI at or above the 95th percentile, following the WHO’s growth reference standards for children and adolescents aged 5–19 years [30].

**Fig 1:**
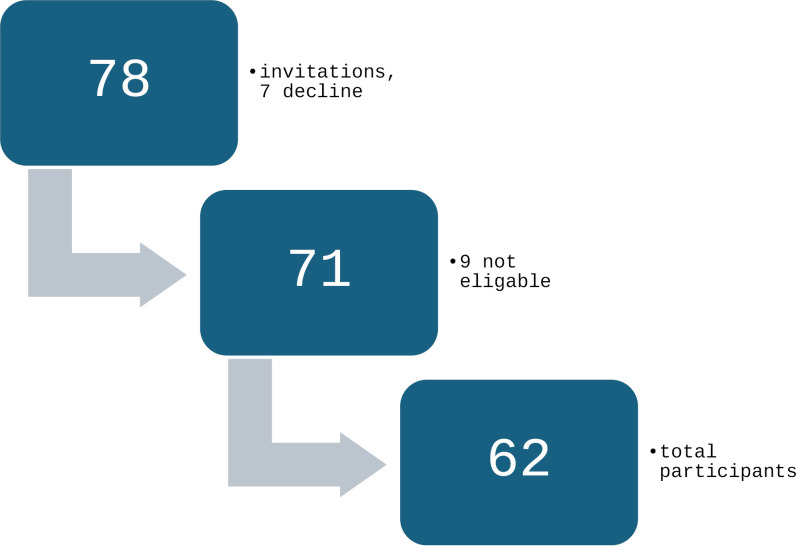
Recruitment of sample flow chart.

A total of 78 adolescents and their parents or guardians were invited to participate, of which seven declined to participate, while a further nine did not meet the eligibility criteria for being overweight or obese. In total, sixty-two participants and their parents provided written consent and assent and agreed to be interviewed (Fig 1). The interviewer established each participant’s BMI classification at the time of the interview.

Participants were stratified by age group (12–15 years and 16–18 years) and by gender to capture perspectives across different stages of adolescence. The aim was also to balance the sample between students enrolled in public and private schools, regions of Saudi Arabia, and rural and urban residences to reflect variation in school environments across Saudi Arabia.

### Strategies for mitigating bias and ensuring objectivity

That the interviewers’ backgrounds in public health education shaped both the questions asked and how they were asked, and how they were interpreted, was acknowledged. Therefore, interviewers received formal training in qualitative methods and cultural sensitivity to minimize interviewer bias. Interviews were also conducted by gender-matched researchers when appropriate. To minimize interpretive bias, transcripts were cross-verified by an independent coder. Verbatim responses were documented to ensure authenticity and reduce interpretative bias. Interviewers were debriefed weekly to review emerging themes and reflect on positionality, and reflexive discussions were held throughout data collection and analysis to explicitly note assumptions made and prioritize adolescents’ voices in shaping the themes. In addition, all transcripts were indpendently coded by two members of the research team, achieving approximately 85% initial agreement. Discrepancies were resolved through discussion until coders reached consensus. This process enhanced the credibility and consistency of the thematic analysis.

### Participant recruitment and consent process

Ethical Approval No.: HAP-09–43 was obtained from the Ethics Committee at Northern Border University. Detailed information sheets and consent forms were distributed to parents/guardians and adolescent participants. Parental consent and adolescent assent were required. All consent and assent agreements were collected via paper forms outlining the time requirement, data usage agreement, and other expectations of the study. Participants and their parents were given time to ask any remaining questions. Participants were assured of the confidentiality of their information, the voluntary nature of participation, and their right to withdraw at any time. Interviews were scheduled after receiving consent and assent forms.

### Data collection procedures

Following consent, participants were invited for interviews held in quiet, private rooms at their schools. Permission was obtained to audio-record the intervies, and each interview lasted 30–45 minutes. Field notes were also taken. Interviews were transcribed verbatim, reviewed for accuracy, and anonymized. The researcher and an accredited translator reviewed translations into English for consistency.

### Data management and analysis

Transcripts were entered into Microsoft Excel for organization, coding, and analysis. Excel was chosen over specialized qualitative software (e.g., NVivo, MAXQDA) due to accessibility constraints and the research team’s prior experience using spreadsheet-based thematic analysis. Manual coding was conducted within Excel.

Thematic analysis followed the method outlined by Bree and Gallagher [19], which involved (i) initial familiarization with the data, (ii) generation of initial codes, (iii) identification of broader categories, (iv) reviewing and refining themes, (v) defining and naming themes, and finally, (vi) producing the thematic map with illustrative quotes.

Key phrases and concepts were thus coded and grouped under major themes and subthemes aligned with a conceptual framework based on the literature review. Coding was both deductive (based on research questions) and inductive (based on emerging insights). In Table 1, the major themes and subthemes extracted through thematic analysis, reflecting adolescents’ views on the determinants, prevention strategies, and health implications of obesity in Saudi Arabia, are summarized.

**Table 1:** Conceptual themes and subthemes for perception analysis [20].

Themes	Subthemes
Factors contributing to obesity	Availability/accessibility of unhealthy food
	Cultural beliefs
	Lack of knowledge
	Peer pressure
	Excessive screen time
Preventative strategies for obesity	Individual motivation
	Public awareness
	National support
Education and health system factors	School curriculum
	School policy
	Health policy
	Training
Stigma	People mocking
	Reduced self-esteem
	Psychological issue
Being obese comes with a high risk	Body weight increases
	Increase in non-communicable diseases (NCD)
	The body’s immunity decreases.

### Study rigor

Study credibility was ensured through methodological transparency, expert validation of the interview guide, and pilot testing. Interpretative triangulation was achieved through researcher debriefs, independent coding, and participant verification of transcripts. The research team included experienced public health professionals, bilingual interviewers, and analysts trained in qualitative methods. Peer debriefings and field note reviews helped ensure the dependability and consistency of findings.

### Ethical considerations

All procedures complied with the Declaration of Helsinki and GDPR. Participant data were securely stored and anonymized in all reporting. Northern Border University’s ethics committee granted ethical approval. Prior to participation, parental consent and adolescent assent were obtained. Additional approvals were secured from school principals and local authorities.

Due to the sensitive nature of adolescent health data and the risk of deductive disclosure, full transcripts cannot be made publicly available. However, for transparency, a summary of the data gleaned for the six main questions is provided in S1 File. Anonymized excerpts relevant to the study findings may be obtained upon reasonable request to the corresponding author (via email), and requests are subject to Ethics Committee review and approval.

## Results

### General characteristics of participants

In Table 2, the demographic data of participants are tabulated.

**Table 2:** Participants Demographic data.

Characteristics	*n*	%
School distribution		
Public	33	53.2
Private	29	46.8
Regional distribution		
Northern	26	41.9
Central	20	32.3
Eastern	14	25.8
Living environment		
Rural	38	61.3
Urban	24	38.7
Age distribution		
12 years	10	16.1
13 years	7	11.3
14 years	8	12.9
15 years	9	14.5
16 years	10	16.1
17 years	9	14.5
18 years	9	14.5
Gender distribution		
Male	30	48.4
Female	32	51.6
BMI (kg/m^2^) distribution		
Mean ±SD	27.82 ± 1.19	

Participants were drawn from both public schools (53.2%) and private schools (46.8%); 41.9% were from the Northern region, 32.3% from the Central region, and 25.8% from the Eastern region of Saudi Arabia; and 61.3% resided in urban and 38.7% rural areas.

The gender distribution among participants was almost equal, comprising 48.4% males and 51.6% females. Distribution by age included 16.1% aged 12, 11.3% aged 13, 12.9% aged 14, 14.5% aged 15, 16.1% aged 16, 14.5% aged 17, and 14.5% aged 18. Participants in this study presented a mean body-mass index (BMI) of 27.82 ± 1.19 kg/m².

### Themes- and subthemes-based analysis

A theme-based analysis guided by the work of Bree and Gallagher [19] was conducted to explore participants’ perceptions of the dietary contributors to excessive weight and obesity.

### Theme 1: Factors contributing to obesity

When asked about the underlying causes of obesity, over half of the participants (*n* = 32, 51.6%) cited the *widespread availability and accessibility of unhealthy food* as a major contributor. *Cultural beliefs* relate to shared societal norms and traditional views about body size and eating, and were mentioned by six participants (9.7%), and 19.4% (*n* = 12) identified a *lack of nutritional knowledge*, either personal or within their families, as a key factor influencing excessive weight gain. *Lack of knowledge* refers specifically to insufficient awareness or understanding of healthy nutrition and lifestyle practices (Table 3).

**Table 3:** Opinion distribution for factors contributing to obesity.

Contributing factors	*n*	%
Easy availability of unhealthy food	32	51.6
Cultural beliefs	6	9.7
Lack of knowledge	12	19.4
Peer pressure	7	11.2
Excessive screen time	5	8.1
Total	62	100.0

A total of 32 participants (51.6%) cited the *availability and accessibility of unhealthy fast food* as a significant factor contributing to their obesity. Among them, six explained that financial constraints limited their ability to purchase healthy foods, such as fresh produce, which they perceived as too expensive. For example, a 13-year-old male noted, “Healthy food is too expensive for my family.” These participants identified the cost of nutritious food as a significant barrier to healthy eating. Many noted the affordability and convenience of energy-dense, nutrient-poor options, such as processed foods high in saturated and Trans fats. Some highlighted the influence of fast food accessibility, explicitly referencing the low cost and ease of obtaining meals from drive-through restaurants (*n* = 6), food delivery apps (*n* = 9), and school canteens (*n* = 2), which increased their frequency of fast-food consumption. Two other participants did not elaborate but cited their family’s low income as the primary cause of unhealthy eating.

Six participants (9.7%) reported *cultural beliefs* as contributing to their weight gain. Two adolescents explained that within their families, meeting their mothers’ expectations for “eating properly” meant overeating, which was normalized within their households and broader Saudi society. Several participants (*n* = 7) described a prevailing belief in their family that “Being overweight means you are healthy,” and as a sign of prosperity (*n* = 5), suggesting familial reinforcement of overnutrition. The remaining two did not provide specific details but referenced cultural norms as influential in their weight gain.

Twelve participants (19.4%) attributed their obesity to a *lack of knowledge* regarding nutrition and healthy lifestyles. Four noted that although their parents may have possessed basic nutritional knowledge, they struggled to apply it effectively to ensure proper feeding practices. Three participants reported that both they and their parents lacked adequate understanding of how to maintain a healthy body weight. The remaining two stated that nutritional awareness was lacking at the community level, resulting in unhealthy dietary patterns from a young age.

*Peer pressure* was another theme mentioned by seven participants (11.2%). Two explained that frequent eating in social groups without accompanying physical activity led to significant weight gain during their school years. Another two recalled that their high school experiences involved prolonged periods of inactivity and group snacking, which contributed to their obesity. Two participants specifically mentioned being introduced to carbonated sugary beverages by friends, which established poor dietary habits that persisted over time.

Last, five participants (8.1%) emphasized *excessive screen time* as a factor influencing their weight gain. Two stated that their daily routines revolved around studying, watching movies, and using mobile devices, leaving little time for physical activity. One participant reflected on the total hours spent in front of screens as “disappointing” and a major contributor to their condition. Yet another shared that they often stayed up late engaging with social media or entertainment, ultimately adopting a sedentary lifestyle that facilitated weight gain.

Gender and age differences were evident in these themes. Female participants more often cited cultural beliefs and peer pressure as influencing their eating behaviors, particularly in relation to body image and family expectations. Younger adolescents (12–15 years) tended to attribute their weight to external factors such as parental control over meals, while older adolescents (16–18 years) more often highlighted lack of knowledge and personal dietary choices.

### Theme 2: Preventive strategies for obesity

This theme encompassed the roles of individual motivation, public awareness, and national support in the prevention of obesity. Both prior research and findings from the present study support the conclusion that prevention is not only more cost-effective than treatment, but also more psychologically manageable for adolescents and families. Addressing the root causes through proactive measures is less discouraging than dealing with the long-term health and emotional consequences associated with being overweight and obese.

Among the participants, 35.5% (*n* = 22) identified individual motivation as the most effective strategy for obesity prevention, 37.1% (*n* = 23) emphasized the importance of public awareness, and 27.4% (*n* = 17) highlighted the need for national-level support and infrastructure (Table 4). These insights reinforce the value of integrating multi-level interventions that include personal, social, and policy-driven components to address the rising burden of adolescent obesity.

**Table 4:** Opinion distribution regarding preventative strategies for obesity.

Preventative strategies	*n*	%
Individual motivation	22	35.5
Public awareness	23	37.1
National support	17	27.4
Total	62	100.0

*Individual motivation* was a factor for 22 participants (35.5%). Sixteen of these participants reported that their motivation to be a healthy weight was lacking, identifying it as a key barrier to weight management. Adolescents shared that poor time management and academic pressure limited their ability to prioritize healthy habits. Four participants emphasized the need for collective efforts or peer-driven initiatives to foster healthier lifestyles. Three respondents stated that they were aware of the importance of engaging in physical activity and reducing food intake, but required encouragement and external accountability to take action. Two participants expressed a desire for motivational support from professionals with backgrounds in physical health and sports. Similarly, another two participants underscored the value of having professional health motivators embedded in school environments. The remaining participants, although unable to elaborate, agreed that personal motivation was critical for achieving and maintaining a healthy weight.

Twenty-three participants (37.1%) identified public awareness as a critical obesity prevention strategy. Of these, four participants suggested that the authorities should launch national awareness campaigns to educate the public and reduce the social stigma associated with obesity. Three participants called for greater involvement from the health department, particularly through community outreach programs designed to inform families about the causes and consequences of weight gain. Two respondents suggested that educational teams should carry out similar awareness-building efforts in schools. Another two participants recommended hosting community-based gatherings in public venues to openly discuss issues related to excess weight and promote healthy living. The remaining participants endorsed the need for public awareness initiatives, although they did not provide specific suggestions.

Finally, national support was emphasized by seventeen participants (27,4%) as an essential component in addressing adolescent obesity. Three participants recommended that the government invest in building more parks, gymnasiums, and playgrounds, accompanied by regular monitoring to ensure these spaces are used effectively. Two participants proposed developing a structured system to assess and support adolescents’ weight and health status through schools or public health initiatives. Another two participants stressed the importance of increased government funding to bolster extracurricular activities that encourage physical movement and healthier routines. The remaining participants acknowledged the importance of national support but did not provide further elaboration. In this theme, older adolescents were more likely to emphasize individual motivation and self-responsibility, while younger participants often stressed public awareness campaigns and external encouragement. No substantial gender difference was observed in preferences for preventative strategies.

### Theme 3: Education and health system aspects

This theme constituted four subthemes: school curriculum, school policy, health policy, and training (Table 5).

**Table 5:** Opinion distribution regarding teaching and health system aspects.

Subthemes	*n*	%
School curriculum	8	12.9
School policy	21	33.9
Health policy	18	29.0
Training	15	24.2
Total	62	100.0

In this study, a third of participants noted that weaknesses in school policy (33.9%) and health policy (29.0%) contribute to adolescent obesity. In addition, nearly a quarter of the participants (24.2%) mentioned training issues and a further 12.9% curriculum issues.

A total of eight participants expressed their opinions that their “school curriculum” contributed to their obesity. Four participants emphasized that evaluating the curriculum for education at primary to secondary levels should include necessary health awareness. Three participants highlighted the importance of including chapters in their textbooks on maintaining good health. Another three participants advocated for mandatory physical education and certification in the curriculum. Two participants mentioned the necessity of including sports in their curriculum. The remaining participants only mentioned the need to develop their school curriculum.

Among our participants, 21 (33.9%) reported that school policy contributed to their obesity. Five participants stated that their school policies were inadequate, with their schools being unclean and not conducive to health. Four participants mentioned that some school policies were outdated and needed to be reviewed to meet contemporary living standards, and three emphasized the need for proper school feeding programs to be included in school policy.

Eighteen participants (29%) expressed their opinions regarding the health policies of their educational institutions. Most (*n* = 6) stated that they were unaware of any health policies at their schools, noting that such policies should be transparent and clear to all students. Three participants reported that while their schools had health policies, these policies primarily addressed issues like transport, alcohol, and smoking. The remaining two participants suggested that their schools should develop modern health policies.

Fifteen participants (24.2%) believed that teachers should be trained in adolescent obesity. Of these, 11 participants highlighted the need for proper training and implementation of appropriate health activities across their educational institutions. Four suggested that sufficient staff should be appointed to manage health issues and that these staff members should receive proper training. The remaining participants mentioned the need for training but did not provide specific details. Male participants more often criticized school policy and the lack of sports facilities, whereas female participants focused on curriculum content related to nutrition and health. Older adolescents were more likely to identify gaps in health policy, suggesting more structured approaches to school-based health promotion.

### Theme 4: Stigma

The three subthemes related to stigma, namely, scornful people, reduced self-confidence, and mental health effects (Table 6). Stigma about excessive weight and obesity is prevalent in this region.

**Table 6:** Opinion distribution regarding stigma.

Stigma	*n*	%
People mocking	19	30.7
Decreased self-esteem	23	37.1
Psychological issues	20	32.2
Total	62	100.0

In evaluating the adverse effects of stigma, 30.7% (*n* = 19) of the participants reported mocking from others; 37.1% (*n* = 23) mentioned decreased self-esteem, and 32.3% (*n* = 20) mentioned psychological issues.

Nineteen participants (30.7%) mentioned people mocking as a form of stigma. Of these participants, eight adolescents reported hearing demoralizing comments while jogging or exercising. For example, a 15-year-old female described, “My uncle always laughs and says I’m too lazy to lose weight, it makes me not want to try anymore.” Three participants claimed they were mocked by family members while trying to manage their obesity through exercise. Two participants reported that their habit of regular exercise became a matter of gossip in their community. The remaining participants identified people mocking them as a barrier to managing their obesity.

Decreased self-esteem was also prevalent among participants. Fourteen participants identified it as a factor influencing their obesity and its management. Eight participants reported that people mocking them was the leading cause of their decreased self-esteem. In addition, three participants highlighted a lack of proper infrastructure in their schools, “because it makes me feel worse about myself” (male, 14 years), and the remaining three participants mentioned insufficient facilities in their community as contributing to their decreased self-esteem.

Twenty participants reported various psychological issues contributing to their obesity. Three participants stated that they felt too shy to exercise. Another three mentioned mental stress as a factor affecting their ability to maintain proper body weight. The remaining six participants articulated that schoolwork, homework anxiety, and other mental stresses were the main barriers to managing their body weight effectively. Female participants more frequently reported stigma related to appearance, reduced self-esteem, and teasing from peers or family, whereas male participants were more likely to view excess weight neutrally or even as a sign of strength. Younger adolescents tended to internalize mocking as a source of shame, while older adolescents discussed its psychological effects in the context of motivation for change.

### Theme 5: Being obese comes with an increased risk of other health problems

Three subthemes emerged from this theme: increased body weight, a rise in non-communicable diseases (NCDs), and decreased immunity (Table 7).

**Table 7:** Opinion distribution regarding ‘being obese comes with high risk’.

High risks	*n*	%
Body weight increases	21	33.9
NCDs increase	25	40.3
The body’s immunity decreases	16	25.8
Total	62	100.0

More than one-third of participants (*n* = 25; 40.3%) believed the risk of non-communicable diseases (NCDs), such as hypertension, diabetes, and cancer, increases with obesity. In addition, 33.9% (*n* = 21) of participants specifically associated obesity with excessive body weight, and 25.80% (*n* = 16) noted that it leads to decreased immune function.

Thirteen participants expressed a shared sense of frustration and resignation about their condition, frequently repeating sentiments such as, “My body weight increases and increases; it seems like a non-stop process” (Female, 18 years). Four participants described feelings of sadness and discouragement, expressing doubts about ever being able to return to a healthy weight. Three reported that weight gain had led them into more sedentary routines, decreasing their physical activity and reinforcing a cycle of inactivity. Two participants said they had lost hope entirely, feeling that sustained weight gain was inevitable and irreversible.

Nineteen participants made various observations linking obesity to NCDs. Among them, four explicitly stated that NCDs are becoming increasingly common and viewed obesity as a significant contributing factor. Three others feared developing chronic diseases due to their weight status. The remaining participants acknowledged a correlation between obesity and NCDs, although they did not express a definitive understanding of causality.

Ten participants also associated obesity with declining immunity. Half believed that their immune systems were weaker than those of their peers with healthy weights. Two participants worried that their immunity was deteriorating over time, while another two believed that low immunity itself might have contributed to their weight gain. One participant attributed their obesity to a specific immunosuppressive medication, suggesting that compromised immunity preceded and possibly triggered weight gain. Older adolescents more often recognized the link between obesity and long-term health risks such as non-communicable diseases, whereas younger participants focused primarily on immediate physical changes such as weight gain or reduced stamina. Gender differences were minimal for this theme.

### Demographic influences

An observational analysis of the interview data shows notable gender-based differences in perceptions of obesity. Female participants more often reported concerns about their bodies, experiences of weight-related social teasing, and pressure from family members regarding their appearance. Many stated emotional distress related to societal appearance standards. In contrast, male participants often perceived being overweight as less problematic, with some relating it to strength or expected growth, and were generally less mindful of long-term health implications.

Age-related patterns were also apparent. Adolescents aged 16–18 years were inclined to show greater mindfulness of the health risks associated with obesity and were more inclined to discuss personal responsibility, dietary habits, and long-term concerns. Younger adolescents (aged 12–15 years) more commonly attributed their weight to external factors such as parental control over meals or lack of physical activity, reflecting a more dependent viewpoint. These demographic discrepancies provide further perspective on the thematic findings.

## Discussion

Helpful insights into Saudi adolescents’ perceptions of dietary contributors to obesity and the strategies they believe are most effective for prevention are provided by this study. Reflecting global patterns, the most frequently cited factor contributing to obesity was the *easy availability of fast food* (51.6%). This finding aligns with prior studies emphasizing how food environments heavily influence dietary choices, particularly among youth [21,22]. Adolescents described fast food as not only convenient but also more affordable than healthier alternatives, which is a concern echoed in recent literature on nutritional inequality and food deserts.

In addition, 19.4% of participants identified a *lack of knowledge* as a primary driver of their weight status. This theme is consistent with international findings showing that poor health literacy significantly impedes adolescents’ abilities to make informed dietary decisions [23]. Moreover, inadequate parental guidance, either due to misinformation or cultural beliefs, was frequently mentioned. In line with Díaz et al.’s ideas, cultural norms that equate larger body sizes with health and prosperity may inadvertently encourage overeating in some families, especially in societies undergoing nutritional transitions like Saudi Arabia [24]. In the Saudi cultural context, family expectations and collectivist meal patterns may reinforce overconsumption, particularly at social gatherings and religious occasions. Gender segregation in physical activity spaces and modesty norms may also limit opportunities for adolescent girls to engage in structured exercise, which is less frequently cited as a barrier in studies from non-segregated societies.

When asked about prevention strategies, *public awareness* emerged as the most common response (37.1%), followed by *individual motivation* (35.5%) and national support (27.4%). These results echo the findings of Teixeira and Marques, who argue that internal motivation is crucial for sustainable health behavior change [25]. However, motivation alone may not be sufficient in the absence of social and systemic support. As highlighted by Joseph et al., adolescents’ dietary decisions are often influenced by psychosocial factors such as stress and peer norms, underscoring the need for comprehensive and contextually appropriate interventions [26].

Indeed, participants pointed to peer influence and social exposure, especially through digital media and group behaviors, as significant barriers to change. This observation is supported by research showing that social eating behaviors and media messaging strongly influence consumption patterns among teens [5]. These findings reaffirm the importance of targeting both the individual and their broader environment when designing intervention programs.

In terms of structural influences, close to half of the participants cited weaknesses in school curriculum (12.9%) and policy enforcement (33,9%), while the remainder raised concerns about health policy gaps (29,0%) and insufficient staff training (24,2%). These findings align with calls for more robust, school-based obesity prevention programs [11,27,28]. The literature suggests that when nutrition and physical education are embedded into formal curricula, students are more likely to develop lifelong healthy behaviors. Policy actions could include revising national school meal standards to reduce sugar and saturated fat content, mandating daily physical education across all grades, providing dedicated training for school staff on adolescent nutrition and mental health, and funding safe, accessible community recreational facilities. The Kiribati study by Tong et al. similarly emphasizes the necessity of school infrastructure, teacher preparedness, and curriculum integration in combating adolescent obesity [11].

Experiences of stigma and its psychosocial consequences were also commonly reported. Some 37.0% and 32.3% of participants, respectively, reported lowered self-esteem and psychological distress, and 30.7% described being mocked. This rate of weight-related teasing is comparable to the findings reported by Puhl and Heuer in Western settings, underscoring the universality of weight stigma across cultural contexts [29]. However, in the current study, teasing often originated within family circles as well as among peers, suggesting that the Saudi context may involve different interpersonal dynamics than those described in a Western context. These findings mirror those in Puhl and Heuer and Stevens et al., who documented the long-lasting effects of weight-based bullying and social exclusion across educational and healthcare settings [20,29]. Participants in this study cited stress, more than depression or bullying, as the primary mental health burden, highlighting the emotional toll of internalized stigma and social pressure.

When discussing health risks, 40.3% of adolescents recognized obesity as increasing the likelihood of developing non-communicable diseases (NCDs), including cardiovascular conditions, diabetes, and hypertension. Other concerns included continued weight gain (33.9%) and weakened immunity (25.8%). These insights are consistent with epidemiological findings by Piernas et al. and Zhang et al., who note that children and adolescents with obesity often show early risk markers for chronic illness [2,30]. Some participants even linked immunosuppression to their own weight gain, either through medication or as a byproduct of unhealthy routines.

An encouraging finding was that several participants mentioned self-imposed behavior change and a positive mindset as essential for managing their health, echoing the findings of Teixeira and Marques, who underscore the power of readiness to change when supported by enabling environments [25]. These findings suggest that adolescents are not only aware of the risks they face but are also open to action, provided they receive the appropriate motivation, education, and structural support.

## Limitations of the research

This study included some limitations. First, it was based on a single round of interviews with a sample of 62 participants within a limited nine-week timeframe. As such, the findings may not fully capture the diverse experiences and perceptions of Saudi adolescents in general. While purposive sampling and regional representation enhance the study’s relevance, caution must be exercised when generalizing results at the national level. In addition, self-reported responses may be subject to social desirability bias, especially regarding sensitive topics such as body image or lifestyle behaviors. The cross-sectional nature of this study also limits examination of how perceptions and behaviors may change over time; future longitudinal research may prove valuable to explore these dynamics.

## Conclusions

Adolescents in this study consistently identified the widespread availability and accessibility of fast food and a lack of nutritional knowledge as central contributors to excessive weight and obesity. They also emphasized systemic shortcomings, including weaknesses in school curricula, inadequate public policies, and insufficient health system support. Stigma emerged as a significant theme, with being mocked by peers reported as the most common form of psychosocial distress, highlighting the emotional and social toll of adolescent obesity. Of note, half of the participants recognized obesity as a risk factor for non-communicable diseases (NCDs), while many cited individual motivation as the most effective strategy for prevention.

These findings underscore the pressing need for a comprehensive, youth-centered approach to obesity prevention, one that addresses not only behavioral factors but also the broader sociocultural and institutional environment in which adolescents live. Public health interventions must extend beyond education to include curriculum reform, anti-stigma campaigns, community engagement, and system-wide policy changes. Empowering adolescents with accurate, accessible information, while creating supportive environments that promote healthy choices, is essential to reversing current obesity trends. For prevention strategies to succeed, the strategies must integrate personal agency with collective responsibility, fostering both self-efficacy and structural support to achieve lasting change.

## Supporting information

S1 File Data perception. (XLSX)
